# Coaxial Laser Cladding of Novel Wear-Resistant Alloy Coatings on 60CrMnMo Steel Surface

**DOI:** 10.3390/ma18204696

**Published:** 2025-10-13

**Authors:** Min Chen, Liu Weng, Xuyang Liu, Zhongxue Feng, Xuan Xiao, Haoran Zhou, Xuefeng Zhang

**Affiliations:** 1 Vanadium and Titanium Critical Strategic Materials Key Laboratory of Sichuan Province, Panzhihua University, Panzhihua 617000, China; cmrre@163.com (M.C.); eiyoggxiaoweng@163.com (L.W.);; 2 Sichuan Vanadium & Titanium Industrial Technology Institute, Panzhihu 617000, China; 3 College of Aerospace Engineering, Chongqing University, Chongqing 400044, China; 4 Faculty of Materials Science and Engineering, Kunming University of Science and Technology, Kunming 650093, China

**Keywords:** coating, laser cladding, Nb content, microstructure, wear resistance

## Abstract

Fe–Cr–Nb–Al–C alloy coatings were firstly fabricated on a high-carbon forged steel surface via coaxial laser cladding. The morphological evolution with varying Nb contents and wear mechanisms of the coatings were systematically investigated through comprehensive analysis. The results indicate that Nb content critically governs the coating microstructure and mechanical properties. At low Nb levels, coarse grain-boundary networks of (Fe,Cr) solid solution embrittled by Cr_23_C_6_ are formed. Moderate Nb addition produces finely dispersed rod-shaped NbC precipitates. At higher Nb levels, the morphology evolves into a segregated martensite–ferrite dual-phase structure. The coating wear rate exhibits a parabolic dependence on Nb content, initially decreasing with moderate addition and then increasing with further Nb. Consequently, optimal wear resistance is achieved at a critical Nb content (3 wt.%) that establishes an optimal balance between NbC precipitation and phase uniformity, thereby minimizing debris generation and spalling.

## 1. Introduction

Carbon steels are preferred structural materials for engineering components due to their favorable mechanical properties, processability, and cost-effectiveness. However, their low surface hardness promotes frictional degradation. Laser cladding of iron-based wear-resistant coatings addresses this limitation by extending service life while reducing resource consumption [1,2,3]. This technique offers high energy density [4], a narrow heat-affected zone [5], rapid interfacial metallurgical bonding [6], and cooling rates exceeding 10^6^ K/s [7,8] that enable unique non-equilibrium microstructures [9].

Extensive research is currently focusing on laser-clad iron–chromium alloy coatings for enhancing carbon steel surface properties. Chromium carbides ((Cr, Fe)_7_C_3_, (Cr, Fe)_23_C_6_)) have been shown to significantly improve wear resistance. However, their coarse grain boundary aggregation induces intergranular embrittlement [10,11,12,13]. Under impact/heavy loads, the aggregation of chromium carbides at grain boundaries often leads to their spalling from the matrix [14,15]. The spalled chromium carbide particles become embedded within the frictional surface layer, significantly accelerating coating wear [12,15,16]. Controlling carbide morphology and distribution at grain boundaries has been demonstrated as an effective approach to mitigate intergranular brittleness in high-chromium iron-based alloys. Liu et al. [15] introduced Ti to form heterogeneous TiC nucleation sites that suppress primary (Cr,Fe)_7_C_3_ growth, simultaneously increasing austenite content and promoting eutectic (Cr,Fe)_7_C_3_ formation, thereby enhancing toughness. Zhang et al. [17] fabricated in situ NbC-reinforced iron-based composite coatings using pure carbon (C), Cr_3_C_2_, Nb, and stainless steel powder, and near-spherical NbC particles were synthesized with a refined grain size and enhanced hardness, wear resistance, and corrosion resistance [9]. Hu et al. [18] systematically investigated the effect of Al on the microstructure and properties of Fe–Cr–C laser-clad coatings on 316L substrates. Their results demonstrated that Al addition reduced the average grain size from 18.6 μm to 6.8 μm by enhancing thermal conductivity and cooling rate of the molten pool, suppressing the eutectic reaction L→γ + (Fe, Cr)_7_C_3_ while promoting the peritectic reaction L + (Fe, Cr)_7_C_3_→γ + (Fe, Cr)_3_C. This phase evolution significantly increased (Fe, Cr)_3_C content while reducing brittle (Fe, Cr)_7_C_3_ formation, leading to a coating hardness increase from 854.7 HV_0.2_ to 1011.3 HV_0.2_, with a 2.1-fold enhancement in wear resistance, while no brittle Fe–Al intermetallic compounds were formed.

The aforementioned studies confirm that introducing strong carbide-forming elements (e.g., Ti, Nb) during laser cladding significantly modifies the distribution of coarse chromium carbides in the matrix. The preferential precipitation of Ti and Nb carbides, uniformly dispersed as nucleation sites, effectively refines the coating microstructure, thereby enhancing toughness, hardness, and wear resistance. However, most existing work has focused on Fe- or Cr-based coatings in general, while systematic studies on Nb additions in coatings deposited on 60CrMnMo high-carbon steel remain scarce. This substrate, although widely used for its high strength and impact resistance, tends to form excessive carbides during cladding, leading to brittleness and cracking. 60CrMnMo steel is widely used in the manufacture of hot-rolling mill rolls for railway rails. However, the most common failure mode of these rolls is surface damage caused by severe wear, which greatly limits their service life. Therefore, developing wear-resistant coatings on 60CrMnMo steel is of significant industrial importance for improving roll durability and reducing maintenance costs.

In this study, crack-free, wear-resistant Fe-based laser-clad coatings were fabricated on 60CrMnMo steel via a coaxial powder-feeding laser cladding process. The alloy design utilized high-carbon ferrochrome as the primary carbon source along with controlled additions of ferroniobium, ferromolybdenum, ferromanganese, and minor Al for microstructure optimization. While Nb has been extensively investigated in Ni-based [19] and Co-based [20] claddings, its effects in Fe-based coatings on high-carbon substrates such as 60CrMnMo have not been systematically investigated. This work aims to fill that gap by elucidating the mechanisms through which Nb additions refine the microstructure, suppress brittle carbides, and enhance wear resistance in Fe-based claddings, thereby providing new insights into alloy design for steel components under severe service conditions.

## 2. Experimental Materials and Methods

The laser cladding experiments were conducted on 60CrMnMo high-carbon forged steel substrates with dimensions of 200 mm × 200 mm × 20 mm (Tebei’er Metal Materials Co., Ltd., Kunshan, China). The chemical composition provided by the manufacturer is detailed in Table 1. Prior to the laser cladding process, the substrate surfaces were cleaned using an RFL-P300 handheld laser descaler to remove rust and oxides, followed by wiping with ethanol to eliminate surface contaminants. This pre-treatment was performed to prevent the formation of pores, inclusions, and other defects in the clad layers.

The feedstock powders comprised ferroniobium (100 μm, Nb: 65 wt.%, Si:2.67 wt.%, C: 0.07 wt.%, and the rest is Fe), high-carbon ferrochrome (150 μm, Cr: 69.31 wt.%, C: 8.51 wt.%, and the rest is Fe), low-carbon ferrochromium (150 μm, Cr: 68.27 wt.%, C:0.1 wt.%, and the rest is Fe), ferromanganese (150 μm, Mn: 80.62 wt.%, Si:1.35 wt.%, C: 0.61 wt.%, and the rest is Fe), spherical pure iron powder (53~150 μm purity greater than 99.99 wt.%), and ferro-molybdenum (150 μm, Mo: 60.71 wt.%, Si: 0.76 wt.%, C: 0.07 wt.%, and the rest is Fe), with minor pure Al powder additions. All raw material powders were provided by Zhujin Technology Co., Ltd. (Tianjin, China). The powders were blended using a ZX-type double-cone high-efficiency mixer (XYZ Machinery Co., Ltd., Shanghai, China) operated at a rotation speed of 60 r·min^−1^ for 10 h to ensure homogeneous mixing. Subsequently, the mixed powders were dried in a constant-temperature (Thermo Fisher Scientific, Waltham, MA, USA) oven at 120 °C for 2 h. Based on this preparation, four Fe-based alloys (designated as Nb-A, Nb-B, Nb-C, and Nb-D) with varying Nb contents were formulated, and their detailed chemical compositions are listed in Table 2.

The experiments were conducted using an LDF 6000-60 coaxial laser cladding system (Laser Line, Mülheim-Kärlich, Germany). Based on our previous optimization studies and published results [13], a laser power of 2200 W was selected, as it provided a good balance between metallurgical bonding quality and defect suppression. Other parameters included an Ar flow rate of 10 L/min, a laser spot diameter of 4 mm, a scanning speed of 10 mm/s, and a powder feeding rate of 2 r/mm controlled by a gas-atomized powder feeder (RC-PGF-D). Following the experiments, the samples were sectioned into two geometries using wire electrical discharge machining: 10 mm × 10 mm × 10 mm blocks for coating composition analysis, phase identification, and microstructural observation and 30 mm × 30 mm × 5 mm coupons for hardness and wear resistance testing.

The phase evolution with temperature was simulated using JMatPro 7.0.0 thermodynamic software. Phase identification was performed utilizing an X’Pert Powder X-ray Diffractometer (Malvern Panalytical, Malvern, UK; Cu Kα radiation, an operating voltage of 40 kV, a step size of 0.02°, a scan speed of 6°/s, and a diffraction angle ranging from 30° to 100°). The polished coating surfaces were etched with aqua regia for a period of 7 to 9 s. The morphology was then observed using a ZEISS Sigma 500 field-emission scanning electron microscope (SEM; Carl Zeiss Microscopy, Jena, Germany) equipped with an energy-dispersive X-ray spectroscopy (EDS; Carl Zeiss Microscopy, Jena, Germany) detector. The transmission electron microscope (TEM; Carl Zeiss Microscopy, Jena, Germany) analysis, incorporating selected-area electron diffraction pattern (SAED) indexing of the precipitates, was conducted using a JEOL JEM-2100 Plus transmission electron microscope (JEOL USA, Peabody, MA, USA). The microhardness was measured on the cross-sectional side surface using an HV-1000STA tester (200 g load, 10 s dwell; LECO Corporation, St. Joseph, MI, USA). Indentations were made perpendicular to the coating surface at 100 μm intervals, and the average value was taken as the reported hardness result. The tribological performance was evaluated on an MVF-1A tester (Jinan HengXu Testing Machine Techology Co., Ltd., Jinan, China) with a YG6 tungsten carbide ball under an 80 N load, 200 r/min rotational speed, and 30 min duration. The wear rate was calculated according to Equation (1):

(1) $$W_{r}=\frac{M_{a}-M_{b}}{T}$$

where *W_r_* is the wear rate, *M_a_* and *M_b_* are the initial and final mass of specimens, respectively, and *T* is the wear time.

## 3. Results and Discussion

### 3.1. Morphology Analysis

**Figure 1:**
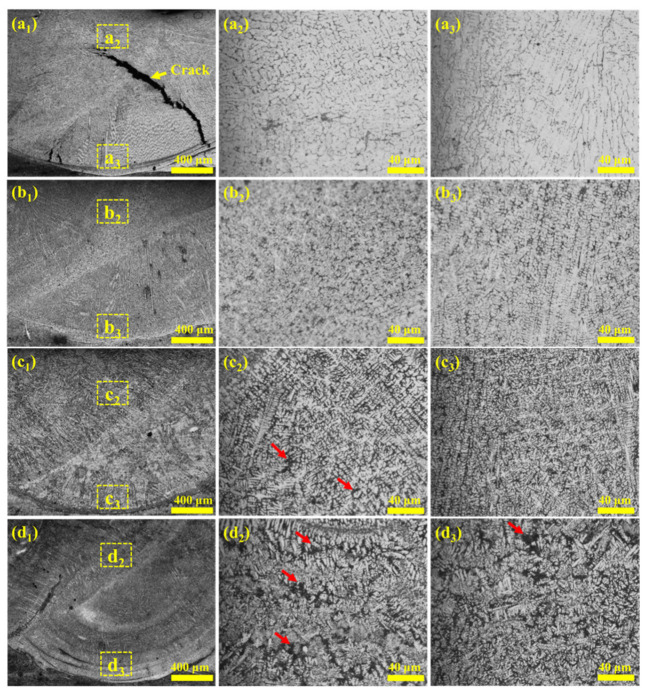
Morphology of the coatings (**a_1_**–**a_3_**): Nb-A, (**b_1_**–**b_3_**): Nb-B, (**c_1_**–**c_3_**): Nb-C, and (**d_1_**–**d_3_**): Nb-D.

Figure 1 illustrates the optical microstructures of the coatings prepared with different Nb contents. Specifically, Figure 1a_1_–d_1_ depict the macroscopic cross-sectional morphologies of the coatings, while Figure 1a_2_–d_2_ and Figure 1a_3_–d_3_ present the corresponding morphology of the coating zone and interfacial bonding zone, respectively. Owing to the rapid heating and cooling characteristics inherent in laser cladding, the coatings exhibit typical non-equilibrium rapid solidification microstructures [21]. Macroscopic cracks are evident in the Nb-A coating with low Nb content (as indicated by the yellow arrows in Figure 1a_1_). The formation of cracks in the Nb-A coating can be attributed to insufficient Nb addition, which fails to effectively suppress the precipitation of brittle phases. At low Nb levels, coarse Cr_23_C_6_ carbides and (Fe,Cr) primary solid-solution phases preferentially segregate along grain boundaries. These brittle constituents induce local stress concentration and act as crack initiation sites [14,22,23]. During subsequent cooling, thermal contraction promotes the propagation of these cracks along weakened grain boundaries, ultimately leading to the formation of macroscopic cracks within the coating. These cracks gradually disappear as Nb content increases. The interface between the coating and the substrate displays a characteristic arc-shaped bonding morphology, evidencing reliable metallurgical bonding. This observation suggests that a higher Nb content effectively mitigates the coating’s brittleness [24]. Furthermore, increasing the Nb content induces significant grain refinement in both the coating zone and the bonding interface [17]. However, segregation occurs in the coating when the Nb content exceeds 5 wt.%, (as indicated by the red arrows in Figure 1).

In Figure 1d_1_, the Nb-D coating exhibits a greater degree of substrate–coating mixing compared to the other samples, despite identical processing parameters. This phenomenon can be attributed to the influence of high Nb content on molten pool dynamics. At elevated Nb levels, the formation of high-melting-point NbC particles and elemental segregation increase the viscosity of the molten pool, thereby suppressing lateral fluid flow. As a result, heat dissipation in the horizontal direction is hindered, and thermal energy becomes more concentrated along the vertical axis. This promotes deeper penetration of the molten pool into the substrate, leading to a higher dilution rate and a larger fraction of substrate material incorporated into the coating [25].

**Figure 2:**
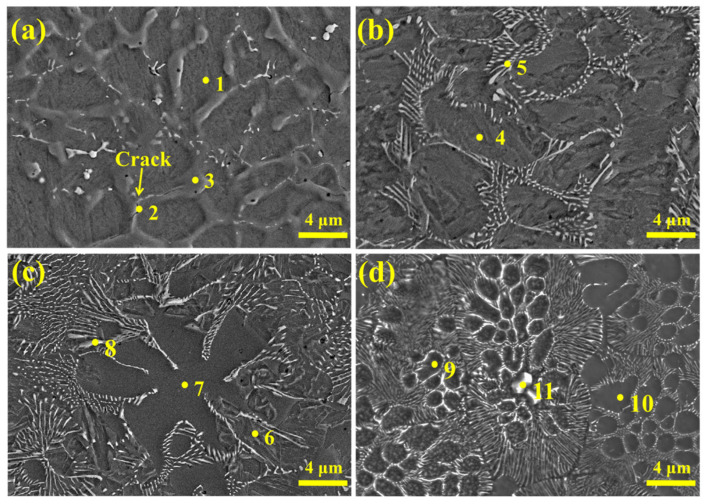
SEM images of the coatings (**a**) Nb-A; (**b**) Nb-B; (**c**) Nb-C; and (**d**) Nb-D.

To examine the effect of Nb content on the morphological evolution of the Fe-based alloy coatings, high-magnification observations of the coating region are presented in Figure 2. At lower Nb contents, a distribution of coarse, skeleton-like primary phases was observed continuously along grain boundaries, forming a network-like structure. Preliminary statistical analysis based on SEM images indicates that, with the increase in Nb content, the area fraction of NbC increases significantly from 2.67% (NB-A) to 6.94% (NB-B), 9.43% (NB-C), and 20.51% (NB-D). With increasing Nb content, these grain boundary skeleton-like phases disappeared, replaced by finely dispersed rod-shaped precipitates. As illustrated in Figure 2c, when the Nb concentration was set at 5 wt.%, the coating matrix exhibited a dual-phase structure, characterized by a martensite–ferrite structure. When the Nb content reached 6 wt.% (Figure 2d), precipitate segregation occurred. It extended from grain boundaries into grain interiors and formed localized honeycomb-like composite structures.

The EDS analysis for the corresponding points in Figure 2 is presented in Table 3. The primary phases constituting the coatings are martensite, ferrite, (Fe,Cr) solid solution, and the carbide phase, which is dependent on the composition of the alloy. The ferrite phase is exclusively present in coatings with a high Nb content, whereas the (Fe,Cr) solid solution is present in coatings with a lower Nb content. EDS analysis further corroborates that the skeleton-like primary phases distributed along grain boundaries are principally (Fe,Cr) solid solutions, while the rod-shaped precipitates are NbC. As a strong carbide-forming element, Nb reacts with carbon in the molten pool to form NbC. This reduces the formation of Cr_23_C_6_ and skeleton-like primary phases, consistent with the refined microstructures observed in Figure 2. The altered grain boundary distribution helps explain the improved toughness of coatings with a moderate Nb content. SEM reveals that increased NbC precipitation preferentially consumed carbon in the molten pool, suppressing the formation of both (Fe,Cr) solid-solution primary phases and Cr_23_C_6_ carbides. The coexisting presence of brittle chromium carbides and skeletal-like primary phases resulted in a substantial weakening of the grain boundary, thereby functioning as nucleation sites for crack initiation.

Elevated Nb levels induce morphological changes in the precipitates. Concurrently, this also facilitates a secondary redistribution of the Cr element within the matrix. As evidenced in Figure 2a–c), the Cr content within the martensitic matrix exhibits an increase from 5.74 wt.% to 5.88 wt.% when Nb contents range from 1 wt.% to 5 wt.%. Localized Cr enrichment is observed in honeycomb-structured regions of the specimen with 6 wt.% Nb content. This phase exhibits a substantially elevated Cr content of 7.05 wt.%, indicative of a significant enrichment. These results demonstrate that the NbC extension from grain boundaries into grain interiors promoted localized Cr enrichment, leading to honeycomb-like segregation.

**Figure 3:**
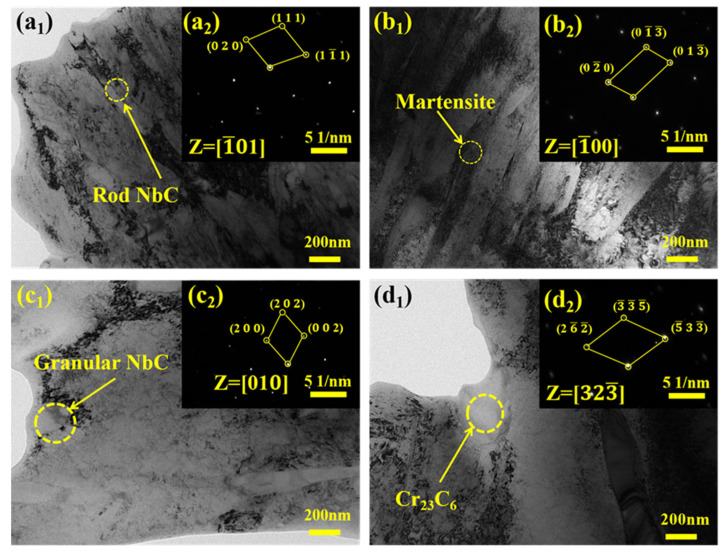
TEM images and SAED patterns of (**a_1_**,**a_2_**) Rod NbC; (**b_1_**,**b_2_**) Martensite; (**c_1_**,**c_2_**) Granular NbC; (**d_1_**,**d_2_**) Cr23C6 in Nb-B coating.

Figure 3 shows the TEM images and SAED diffraction patterns of the Nb-B coatings. Rod-shaped NbC precipitates with a diameter of approximately 40 nm to 50 nm formed on the martensitic matrix. Cr_23_C_6_ carbides are observed to exhibit a face-centered cubic (FCC) structure, with an average size of 250 nanometers. The SAED patterns of NbC demonstrate low-index diffraction spots corresponding to (0 2 0), (1 1 1), and (1 −1 1), thereby indicating a NaCl-type crystal structure with a lattice parameter of approximately 4.47 Å. In contrast, the Cr_23_C_6_ patterns display high-index diffraction spots including (−3 −3 −5), (2 −6 −2), and (−5 3 −3), indicative of its complex FCC structure with a larger lattice parameter of approximately 10.6 Å. The complex crystal structure of Cr_23_C_6_ correlates with its higher brittleness [26], whereas the simpler NbC crystal structure contributes to enhanced toughness.

### 3.2. Phase Analysis

**Figure 4:**
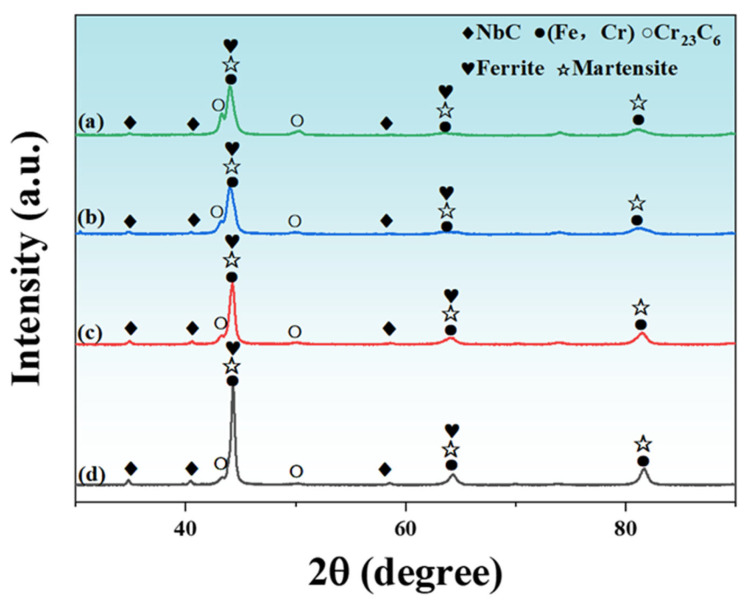
XRD diffraction patterns of coatings (a) Nb-A; (b) Nb-B; (c) Nb-C; and (d) Nb-D.

Figure 4 presents the XRD patterns of laser-clad Fe-based alloy coatings with varying Nb contents. The phase identification further confirms a dual-phase constitution consisting of matrix phases (martensite, ferrite, and (Fe,Cr) solid solution) and precipitated phases (predominantly NbC and Cr_23_C_6_). Owing to the relatively minimal Al content, its phase composition cannot be definitively ascertained by XRD analysis. The X-ray diffraction peaks of the matrix phases exhibit pronounced broadening characteristics upon the addition of 3 wt.% Nb content, indicating significant grain refinement. Metallographic and SEM observations reveal that both Nb-A and Nb-B coatings consist primarily of a martensitic matrix. The synergistic effect of Nb [17] and Al [18] elements significantly refined the grains. Specifically, Nb refined the grains by forming NbC that acted as nucleation sites and pinned grain boundaries, while Al promoted refinement by enhancing molten pool thermal conductivity and cooling rate. With increasing Nb content, the diffraction peak intensity of NbC progressively intensified, while that of Cr_23_C_6_ diminished, forming a ferrite/martensite dual-phase matrix. Concurrently, the carbon-depleted zones around the NbC precipitates facilitated ferrite formation via the ferrite-stabilizing effect of Al [27,28]. After the dual-phase matrix formed, the grain-refining effect of Al diminished. This was evidenced by sharper X-ray diffraction peaks.

**Figure 5:**
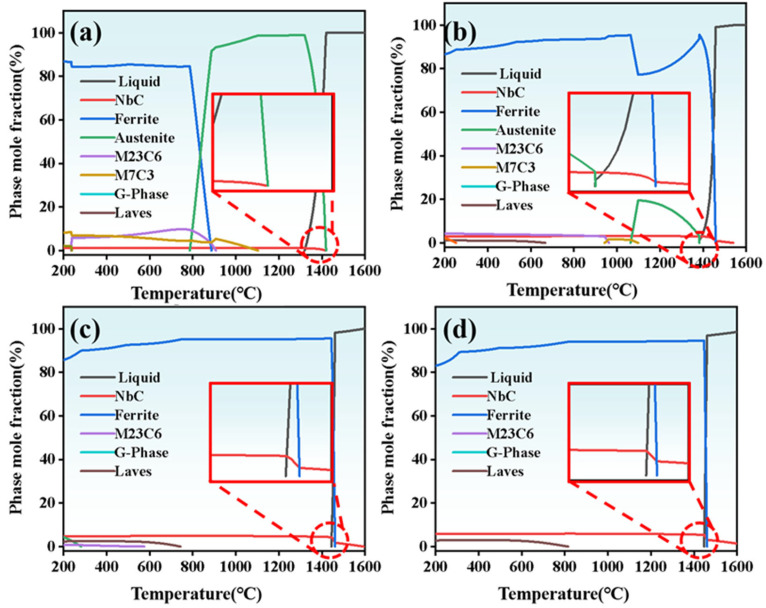
The thermodynamic equilibrium phase diagrams of coatings (**a**) Nb-A; (**b**) Nb-B; (**c**) Nb-C; and (**d**) Nb-D.

Figure 5 depicts the thermodynamic equilibrium phase diagrams of Fe-based alloys with varying Nb contents. Given the rapid solidification inherent to laser cladding, featuring cooling rates ranging from 10^4^ to 10^5^ °C/s [7,8], austenite undergoes direct transformation into a martensitic matrix via a diffusionless mechanism. In the presence of Al, an increase in Nb content results in a rapid shift of phase field from austenite to ferrite, leading to a gradual reduction in the martensite content within the matrix. Thermodynamic equilibrium phase analysis indicates that Cr exists in two carbide forms at low Nb contents: M_23_C_6_ and M_7_C_3_. As Nb content increases, the M_7_C_3_ phase disappears, while the M_23_C_6_ gradually decreases. The experimental results demonstrate that M_23_C_6_ exhibited superior phase stability under these alloying conditions, which was consistent with the phase identification in Figure 4. This suppression of Cr-rich carbides also explains the disappearance of coarse skeleton-like phases at grain boundaries and the emergence of finer NbC precipitates observed in Figure 2. With increasing Nb content, both the amount of precipitated NbC and its precipitation temperature progressively increase, indicating that the incorporation of high-melting-point ferroniobium raw materials elevates the coating’s melting temperature. When Nb content exceeds 3 wt.%, the enlarged view of the primary NbC precipitation zone reveals that NbC and ferrite simultaneously nucleate from the high-temperature liquid phase region, forming a characteristic eutectic microstructure [29,30,31]. As Nb content increases, the M_7_C_3_ phase disappears, while the M_23_C_6_ gradually decreases. This trend is consistent with the microstructural observations: at low Nb levels, coarse Cr_23_C_6_ carbides segregate along grain boundaries, weakening the matrix, whereas at higher Nb contents, fine NbC precipitates form preferentially and become uniformly dispersed within the matrix. The precipitation of NbC consumes carbon and competes with chromium, thereby suppressing the formation of Cr-rich carbides. As a result, the amount of M_23_C_6_ is significantly reduced, and M_7_C_3_ is no longer detected, in agreement with the XRD patterns [24,32]. It should be noted that thermodynamic predictions assume equilibrium, while laser cladding involves rapid non-equilibrium solidification, which may cause deviations from observed phase evolution.

### 3.3. Mechanical Property

#### 3.3.1. Hardness Characterization

**Figure 6:**
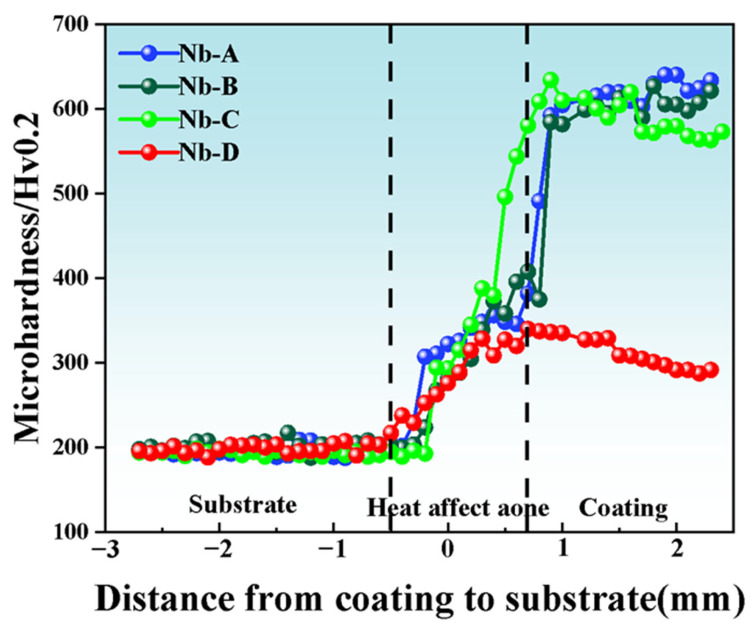
Microhardness distribution curves of the coatings (a) Nb-A; (b) Nb-B; (c) Nb-C; and (d) Nb-D.

Low-magnification metallography reveals an arc-shaped bonding interface between the substrate and coating. The bonding interface exhibits an arc-shaped geometry that is symmetric about its central axis. Therefore, microhardness measurements were performed along this symmetry axis from the substrate–coating interface toward the coating surface. Therefore, microhardness measurements were conducted along this symmetrical axis. The results are presented in Figure 6. Systematic observations reveal a consistent increase in microhardness from the substrate to the coating surface, with this upward gradient persisting across the bonding interface.

The remarkable hardness of the Nb-A coating results from two synergistic mechanisms: solid-solution strengthening by extensive (Fe,Cr) solid-solution phases [22] and dispersion strengthening from Cr_23_C_6_ carbides along grain boundaries [23]. Although these carbides contribute to hardness, their intergranular distribution may also increase brittleness, implying that Nb content must be carefully controlled to achieve an optimal balance between strength and toughness. When the Nb content exceeds 5 wt.%, a noticeable decline in hardness (<400 HV0.2) is observed. This degradation is primarily attributed to excessive NbC precipitation, which not only suppresses the formation of (Fe,Cr) solid-solution phases and Cr_23_C_6_ carbides but also promotes Cr enrichment in the matrix, thereby reducing the driving force for carbide precipitation and increasing ferrite formation. Consequently, the coexistence of soft ferrite and hard martensite phases leads to hardness mismatch, and further Nb addition (e.g., 6 wt.%) aggravates elemental segregation and ferrite fraction, ultimately diminishing hardness. These findings underline the critical role of Nb in regulating precipitate evolution and phase constitution, which collectively determine the hardness–brittleness balance of the coatings.

#### 3.3.2. Wear Performance

**Figure 7:**
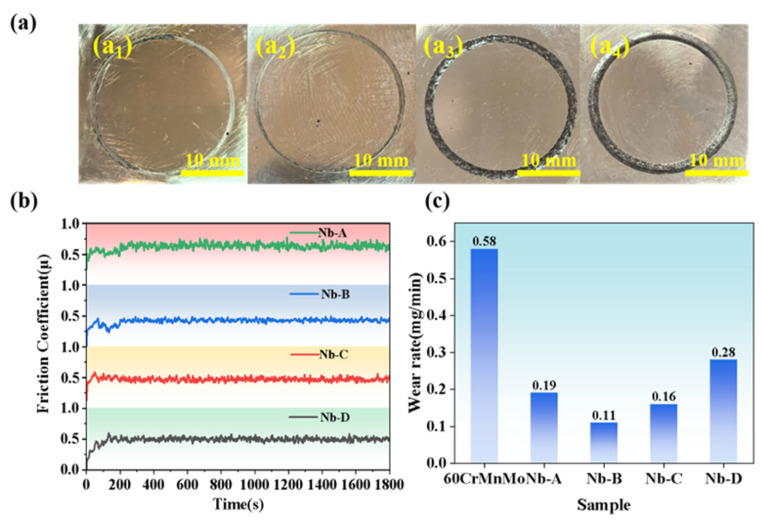
(**a**) Surface morphology: (**a_1_**) Nb-A, (**a_2_**) Nb-B, (**a_3_**) Nb-C, (**a_4_**) Nb-D; (**b**) COF curves; and (**c**) wear rate of coatings after frictional experiment.

Figure 7 shows the wear test results for the four designed coatings. As illustrated in Figure 7a, the Nb-B coating exhibits the shallowest and narrowest wear track, while the Nb-D coating displays the deepest and widest. The wear tracks become more pronounced in depth and width with increasing Nb content, particularly at Nb contents higher than 5 wt.%, indicating a decline in wear resistance. The real-time coefficient of friction (COF) curve and wear rate of coating specimens are presented in Figure 7b,c, respectively. An initial running-in period for both coating and counterpart results in a gradual increase of the friction coefficient.

Microscopic and macroscopic analyses of wear morphology, COF curve, and wear rates reveal that the Nb-B coating exhibits optimal wear resistance, with an average COF of 0.42 and wear rate of 0.11 mg/min. In contrast, the Nb-A coating demonstrates moderate wear resistance (COF = 0.53, wear rate = 0.19 mg/min), which is attributed to increased intergranular brittleness and crack initiation induced by Cr_23_C_6_ precipitates. Under cyclic loading, these cracks propagate, leading to detachment of Cr_23_C_6_ particles that act as third-body abrasives, thereby promoting a transition from a two-body to three-body wear mechanism and concomitant increases in wear rate. At 5 wt.% Nb, the Nb-D coating exhibits a significant increase in both COF and wear volume, accompanied by pronounced COF fluctuations. Microstructural characterization indicates elevated ferrite content in the Nb-D matrix, which undergoes repeated adhesive interactions with the friction pair during wear testing. This adhesive behavior exacerbates abrasive wear mechanisms and diminishes the coating’s wear resistance. In addition, all Nb-modified coatings exhibited markedly improved wear resistance compared with the 60CrMnMo substrate (wear rate: 0.58 mg/min), confirming the effectiveness of laser cladding in enhancing the surface durability of high-carbon steels.

#### 3.3.3. Wear Track Morphology Analysis

**Figure 8:**
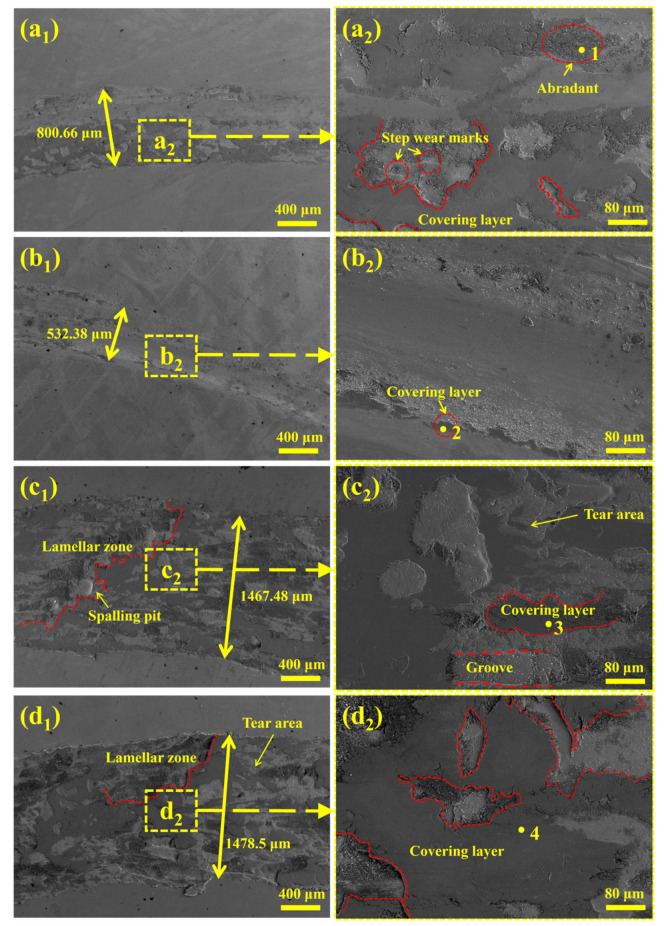
Friction and wear morphologies for specimens with different Nb contents: (**a_1_**,**a_2_**) Nb-A; (**b_1_**,**b_2_**) Nb-B; (**c_1_**,**c_2_**) Nb-C; (**d_1_**,**d_2_**) Nb-D.

The wear track morphologies of the specimens in Figure 8 further corroborate the results in Figure 7a. The high-magnification images in Figure 8a_2_–d_2_ combined with EDS analysis of adhesive wear regions (Table 4) reveal that wear tracks contain trace W and elevated O concentrations, indicating synergistic oxidative, adhesive, and abrasive wear mechanisms between the coating and WC counterface. The surfaces of all coatings form stable oxide films and fine oxide particles, which act as lubricants and stabilize the coefficient of friction.

For the Nb-A coating, all three aforementioned wear mechanisms are operative: initial wear induces microcracks in network-like (Fe,Cr) solid-solution and brittle Cr_23_C_6_ along grain boundaries, which propagate to form tear areas and lamellar zones with stepwise features. Mixed wear debris acts as abrasives, with some compacted into dense layers and others forming grooves via cold welding. In contrast, the Nb-B coating shows reduced adhesive and abrasive wear. The uniformly distributed short-rod NbC precipitates strengthen the martensitic matrix, inhibit detachment, and minimize debris accumulation.

The Nb-C and Nb-D coatings, featuring ferrite/martensite dual-phases, show distinct wear behaviors. As reported by Ouyang et al. [33], non-uniform soft–hard phase distribution generates lamellar zones on the worn surface, with ferrite serving as spalling initiation sites. Repeated adhesion–detachment cycles form grooves and pits, transitioning the wear mechanism to adhesive–abrasive dominance. For the ferrite-rich Nb-D coatings, Figure 8c_1_–d_1_ illustrate extensive lamellar zones and tear areas, while Figure 8c_2_–d_2_ reveal wear pits filled with abrasives and distinct spalling features. The increased ferrite content and non-uniform dual-phase distribution are identified as the primary causes of severe wear, culminating in the highest wear loss observed in Nb-D.

**Figure 9:**
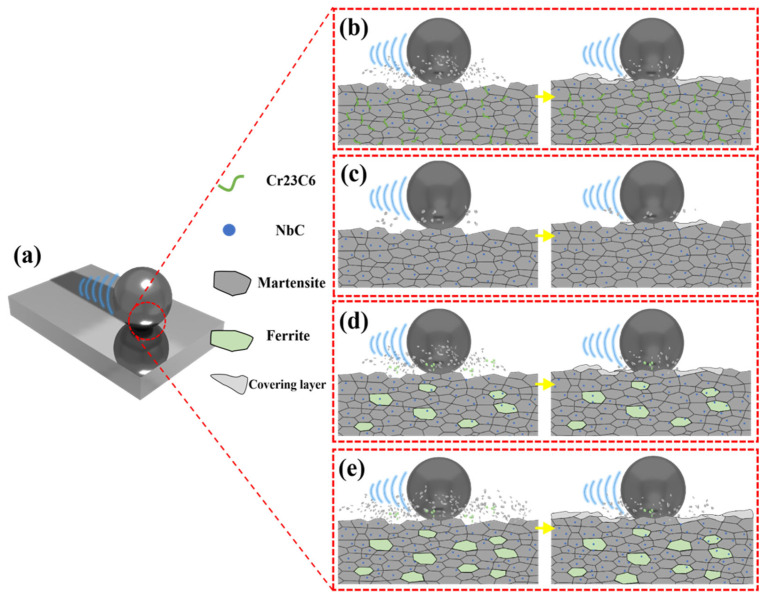
(**a**) Schematic diagram of friction and wear and (**b**–**e**) wear cross-section diagrams of specimens with different Nb contents: (**b**) Nb-A; (**c**) Nb-B; (**d**) Nb-C; and (**e**) Nb-D.

Figure 9 summarizes the above-discussed friction and wear mechanisms of coatings with varying Nb content. The superior wear resistance of the Nb-B coating stems from Nb eliminating brittle Cr_23_C_6_ at grain boundaries and the synergistic strengthening of the martensitic matrix with a dispersed NbC phase. By contrast, the deteriorated wear resistance of the Nb-C and Nb-D coatings arises from excess Nb content, which induces a ferrite/martensite dual-phase matrix in the coating.

The present study focuses on microstructure and wear under laboratory conditions. Long-term wear stability and corrosion resistance, while important for practical applications, were beyond the current scope and will be addressed in future work.

## 4. Conclusions

In this study, Fe-based wear-resistant coatings with varying Nb contents were fabricated on 60CrMnMo high-carbon forged steel via laser cladding. The influence of Nb content on the coating’s microstructure, phase composition, hardness, and wear resistance was systematically examined. The main conclusions are summarized as follows:

1. Insufficient Nb addition (<3 wt.%) leads to a brittle coating microstructure characterized by grain-boundary networks of (Fe,Cr) solid solution and Cr_23_C_6_ carbides. These brittle phases serve as crack initiation sites, resulting in severe abrasive–adhesive wear.

2. An optimal Nb content of 3 wt.% yields the best wear performance, with a wear rate of 0.11 mg/min and a coefficient of friction of 0.42. This improvement is attributed to the suppression of macroscopic cracking, microstructural refinement via uniform NbC precipitation, and reduced debris generation during sliding.

3. Excessive Nb addition (>5 wt.%) promotes the formation of ferrite and causes NbC segregation, resulting in a heterogeneous dual-phase matrix. This microstructure exhibits poor wear resistance due to adhesive material removal and lamellar spalling.

## Figures and Tables

**Table 1:** Elemental composition of 60CrMnMo high-carbon forged steel substrates.

Element	Ni	C	Si	Mn	Cr	Mo	Fe
Composition(wt.%)	1.5~2.0	0.55~0.65	0.2~0.4	0.6~1.0	0.7~1.0	0.1~0.3	Bal.

**Table 2:** Elemental composition of four Fe-based alloy powders (wt.%).

	Fe	Cr	Mn	Si	Mo	Al	Nb	C
Nb-A	Bal.	9.1	2.7	1.05	1.4	2.0	1	1.01
Nb-B	Bal.	9.1	2.7	1.05	1.4	2.0	3	1.01
Nb-C	Bal.	9.1	2.7	1.05	1.4	2.0	5	1.01
Nb-D	Bal.	9.1	2.7	1.05	1.4	2.0	6	1.01

**Table 3:** EDS analysis for the corresponding points in Figure 2 (wt.%).

	Point	C	Al	Cr	Mn	Nb	Mo	Fe	Type
Nb-A	1	6.87	1.86	5.74	1.51		0.65	Bal.	Martensite
	2	8.54	0.89	26.68	1.88			Bal.	Chromium Carbon
	3	4.03	2.28	9.19	2.28		0.89	Bal.	Primary Phase
Nb-B	4	5.81	2.86	5.86	1.69			Bal.	Martensite
	5	8.64	0.46	2.54	1.73	20.38	1.59	Bal.	NbC
Nb-C	6	5.32	1.37	5.88	1.76			Bal.	Martensite
	7	3.63	1.46	6.37	2.28			Bal.	Ferrite
	8	8.34	0.47	2.71	1.79	30.45	1.47	Bal.	NbC
Nb-D	9	4.61	1.74	7.05	2.22	1.33	0.95	Bal.	Segregated Phase
	10	3.51	1.86	6.54	1.69			Bal.	Ferrite
	11	10.89	0.04	1.42		73.5	1.94	Bal.	NbC

**Table 4:** EDS results of relatively smooth adhesive wear areas corresponding points in Figure 8 (wt.%).

Point	C	O	Al	Si	Cr	Mn	Fe	Ni	Nb	W
1	5.19	19.04	1.31	0.36	5.31	1.83	Bal.	1.12	3.93	2
2	5.32	20.87	1.46	0.42	5.43	1.81	Bal.	0.87	2.47	1.9
3	9.63	21.78	1.13	0.39	6.72	1.51	Bal.	0.98	3.23	1.87
4	4.67	18.92	1.28	0.36	6.58	1.45	Bal.	0.93	5.41	2

## Data Availability

The original contributions presented in this study are included in the article. Further inquiries can be directed to the corresponding author.
